# Dynamic Shifts in Lymphocyte Subsets During the Menopausal Transition and Sexual Dimorphism in Immunosenescence

**DOI:** 10.1002/iid3.70558

**Published:** 2026-10-15

**Authors:** Ci Xuan, Li He, Zhou Bohan, Xu Junming, Li Qingchao, Huang Junting

**Affiliations:** ^1^ Department of Blood Transfusion The First Affiliated Hospital of Guangzhou Medical University Guangzhou Guangdong China; ^2^ Department of Emergency Medicine The First Affiliated Hospital of Guangzhou Medical University Guangzhou Guangdong China; ^3^ Nanjing Southeast Research Institute Nanjing Jiangsu China; ^4^ Department of Emergency Medicine, Loudi Hospital The First Affiliated Hospital of Guangzhou Medical University Loudi Hunan China

**Keywords:** Chinese population, immunosenkescence, lymphocyte subsets, menopausal transition, PD‐1, sexual dimorphism

## Abstract

**Background:**

Immunosenescence demonstrates marked sexual dimorphism, yet the dynamic changes in lymphocyte subsets across the female menopausal transition are poorly defined. This study characterized these age‐ and sex‐related immune variations in a large Chinese cohort.

**Methods:**

In this cross‐sectional study, we analyzed 4209 healthy adults using flow cytometry to quantify lymphocyte subsets and PD‐1 expression. Females were stratified by age and reproductive stage. Sex comparisons were performed after propensity score age‐matching.

**Results:**

Analysis of the female cohort revealed dynamic immune shifts during the menopausal transition, characterized by a “scissor‐like” pattern: CD3^+^CD4^+^ T cells (%) and the CD4/CD8 ratio peaked at 55–59 years, while CD3^+^CD8^+^ T cells (%) declined continuously (all *p* < 0.001). Notably, CD19^+^ B cells (%) increased with age in females but decreased in males. After age‐matching, pronounced sexual dimorphism was confirmed: females exhibited significantly higher levels of CD3^+^ T cells, CD3^+^CD4^+^ T cells, CD19^+^ B cells, NK cells, and CD4/CD8 ratio, alongside lower PD‐1 expression on CD4^+^ T cells and NK cells (all *p* < 0.001). In contrast, PD‐1 expression on CD8^+^ T cells was higher in females (*p* < 0.001).

**Conclusion:**

These findings suggest that the perimenopausal period represents a key phase of immune remodeling in women, and support the integration of sex and reproductive stage into future immunosenescence research and clinical strategies.

## 1 Introduction

Immunosenescence is a progressive process characterized by a decline in T‐cell receptor diversity, reduced output of naïve T cells, and the development of a chronic low‐grade inflammatory state known as inflammaging [1]. The characteristics of immunosenescence include a decline in cell‐mediated immune function and humoral immune responses [2, 3]. These age‐related alterations collectively impair immune responses to infections, malignancies, and vaccinations [4, 5]. Notably, the trajectory of immunosenescence exhibits substantial inter‐individual heterogeneity, with sex being recognized as a critical determinant [6, 7].

Extensive epidemiological and clinical evidence demonstrates a pronounced sexual dimorphism in immune responses. Compared with males, females generally mount stronger innate and adaptive immune responses to pathogens and vaccines, but concurrently display a higher susceptibility to autoimmune diseases [8, 9]. These observations suggest that male and female immune systems may undergo distinct aging trajectories. Although previous studies have reported sex‐specific differences in circulating T‐cell subsets and inflammatory markers [10, 11], a systematic and detailed characterization of how these differences evolve across the aging process remains limited.

The female life course is characterized by unique reproductive endocrine transitions, with the menopausal transition representing a critical period marked by rapid hormonal fluctuations [12]. Experimental evidence has established that sex hormones, particularly estrogen and progesterone, exert broad immunomodulatory effects by regulating T‐cell activation, B‐cell differentiation, and the cytotoxic activity of natural killer (NK) cells [13, 14, 15]. Despite this biological plausibility, large‐scale population‐based studies that comprehensively characterize the dynamic trajectories of lymphocyte subsets during the menopausal transition are scarce.

In this study, we aimed to systematically characterize age‐related changes in lymphocyte subsets in a healthy Chinese population, with a particular focus on females. The Stages of Reproductive Aging Workshop +10 (STRAW + 10) system provides a widely accepted framework for staging reproductive aging in women based primarily on menstrual cycle patterns [16]. In this study, we adopted age‐based stratification that approximates the key reproductive phases defined by this framework, allowing us to describe immune profiles across the menopausal transition in a large community‐based cohort. Our primary objective was to characterize age‐ and sex‐related differences in lymphocyte subsets and PD‐1 expression in a healthy Chinese population, with a particular focus on the perimenopausal period in women.

## 2 Materials and Methods

### 2.1 Study Cohort

This cross‐sectional study was conducted to characterize age‐related changes in lymphocyte subsets among women spanning the critical perimenopausal window (40–65 years), with men included as a reference group. Participants were derived from the healthy control cohort of the broader research program “Dynamic Immunoinflammatory Assessment of Early Prognosis in Septicemia” conducted by the First Affiliated Hospital of Guangzhou Medical University (Loudi Hospital), with recruitment and testing performed by the Nanjing Southeast Research Institute. Between January 2022 and December 2023, a total of 4209 adult volunteers were consecutively enrolled, including 2898 women and 1311 men. The study was conducted in accordance with the Declaration of Helsinki and approved by the institutional ethics committee (Approval No. 2026‐010‐01). Written informed consent was obtained from all participants prior to blood sample collection. All personal identifiers were removed, and the data were analyzed in anonymized form.

Participants with active immune‐related disorders, malignancies, acute infections within 4 weeks prior to enrollment, or chronic immunosuppressive therapy were excluded to ensure a stable immune background. In addition, women who were pregnant or breastfeeding at the time of enrollment were also excluded. Female participants were categorized into eight age groups (18–39, 40–44, 45–49, 50–54, 55–59, 60–64, 65–69, and ≥ 70 years). These age intervals were chosen to approximate the key reproductive phases defined by the STRAW + 10 framework, while allowing for sufficient sample size in each stratum for robust statistical comparison.

### 2.2 Flow Cytometry Analysis

Flow cytometry was performed using a FACSCanto II instrument (BD Biosciences, San Jose, CA, USA) to analyze peripheral lymphocyte subsets. For each sample, 50 µL of heparin‐anticoagulated whole blood was aliquoted into two Trucount tubes pre‐mixed with antibody cocktails. Tube 1 contained an antibody cocktail of FITC‐CD4, PE‐CD8, PerCP‐Cy5.5‐CD3, and APC‐PD‐1 for analyzing CD4^+^ T cells, CD8^+^ T cells, and their PD‐1 expression. Tube 2 contained an antibody cocktail of FITC‐CD3, PE‐CD(16 + 56), PerCP‐Cy5.5‐CD19, and APC‐PD‐1 for identifying NK cells and B cells, and for analyzing PD‐1 expression on NK cells. Incubated at room temperature (20°C–25°C) for 15 min in the dark, and drop 500 μL of 1X FACS lysing solution into each tube. The tubes were further incubated for 10 min in the dark at room temperature (20°C–25°C) and then analyzed using the flow cytometer.

Lymphocytes were identified by their strong CD45 expression and low side scatter. Subpopulations including CD3^+^CD4^+^ T cells, CD3^+^CD8^+^ T cells, and CD3^−^CD (16 + 56)^+^ NK cells were further analyzed. The positive threshold for PD‐1 was established using a fluorescence minus one (FMO) control. The percentages were automatically calculated using BD Multitest software (BD Biosciences).

### 2.3 Statistical Analysis

The data were analyzed by SPSS version 26.0 for Windows (SPSS Inc., Chicago, IL, USA). The Kruskal–Wallis test and Mann–Whitney *U* test were used to compare lymphocyte subsets across age groups and between sexes. A value of *p* < 0.05 was considered statistically significant. To determine whether the patterns observed in females were indeed sex‐specific, a rigorous sex comparison was performed. To minimize potential confounding arising from imbalanced age distributions, propensity score matching was used to construct a 1:1 male‐to‐female matched cohort (caliper = 0.01). Within this matched cohort, the Mann–Whitney *U* test was applied to compare differences in each lymphocyte subset between males and females across all age groups.

## 3 Results

### 3.1 Distribution of Data and Elimination of Outliers

T‐lymphocyte subset test results are non‐normally distributed by the Skewness–Kurtness test. To facilitate the identification of outliers, Box‐Cox transformation was applied to each immune parameter using Stata 18.0 (with λ estimated by maximum likelihood), which rendered the data approximately normally distributed (Table 1). We used the Tukey method to eliminate 31, 77, 43, 86, 82, 39, 17, 24, 52, 5 cases of outliers and finally obtained 4178, 4132, 4166, 4123, 4127, 4170, 4192, 4185, 4157, 4204 reference individuals for CD3^+^T cell (% Lymphocytes), CD3^+^CD4^+^T cell (% Lymphocytes), CD3^+^CD8^+^T cell (% Lymphocytes), CD4/CD8 ratio, CD19^+^B cells (% Lymphocytes), CD16^+^56^+^cells (% Lymphocytes), PD‐1^+^ (% of CD4^+^ T cells), PD‐1^+^ (% of CD8^+^ T cells), PD‐1^+^ (% of NK cells), NKT (% of lymphocytes), respectively (Table 1).

**Table 1:** Assessment of Normality Using Skewness and Kurtosis for T‐Lymphocyte Subsets, with Box‐Cox Transformation Parameters (λ) and Outlier Count Based on Tukey's Method.

Immunological parameter	Before BOX‐COX conversion		After BOX‐COX conversion		λ	Outliers removed	N Included
	Skewness	Kurtosis	Skewness	Kurtosis			
CD3^+^T cells (% Lymphocytes)	−0.636	1.106	−0.003	0.037	2.50	31	4178
CD3^+^CD4^+^T cells (% Lymphocytes)	−0.627	1.345	0.026	0.411	0.37	77	4132
CD3^+^CD8^+^T cells (% Lymphocytes)	1.040	1.772	0.009	0.659	0.12	43	4166
CD4/CD8 Ratio	1.634	9.189	0.007	1.373	0.39	86	4123
CD19^+^B cells (% Lymphocytes)	0.520	0.878	0.006	0.559	0.62	82	4127
CD16^+^56^+^cells (% Lymphocytes)	1.237	2.642	0.008	0.157	0.23	39	4170
PD‐1^+^ (% of CD4^+^ T cells)	0.898	1.007	−0.004	−0.103	0.33	17	4192
PD‐1^+^ (% of CD8^+^ T cells)	0.971	1.306	−0.006	−0.056	1.68	24	4185
PD‐1^+^ (% of NK cells)	1.844	8.447	−0.025	0.659	0.36	52	4157
NKT (% of Lymphocytes)	1.685	4.401	−0.017	−0.392	0.16	5	4204

### 3.2 Correlation Between T Lymphocyte Subsets and Age in Different Sex Groups

Analysis of age‐related immune dynamics, stratified by sex, revealed distinct patterns in the female cohort (Figure 1, Table S1). In females, key negative correlations with age were observed for CD3^+^ T cells (%) (*r* = −0.152) and CD3^+^CD8^+^ T cells (%) (*r* = −0.116), whereas CD3^+^CD4^+^ T cells (%) (*r* = 0.043), the CD4/CD8 ratio (*r* = 0.088), and CD19^+^ B cells (%) (*r* = 0.070) showed positive associations. Notably, several sex‐specific divergences were identified. The most striking was observed in CD19^+^ B cells (%), which correlated positively with age in females but negatively in males (*r* = −0.110). Furthermore, the positive association of CD16^+^56^+^ NK cells (%) with age, while present in both sexes, was markedly more pronounced in males (*r* = 0.185) than in females (*r* = 0.106). Complete correlation coefficients and regression slopes for all immune subsets are detailed in Supporting Information S1: Table S1.

**Figure 1:**
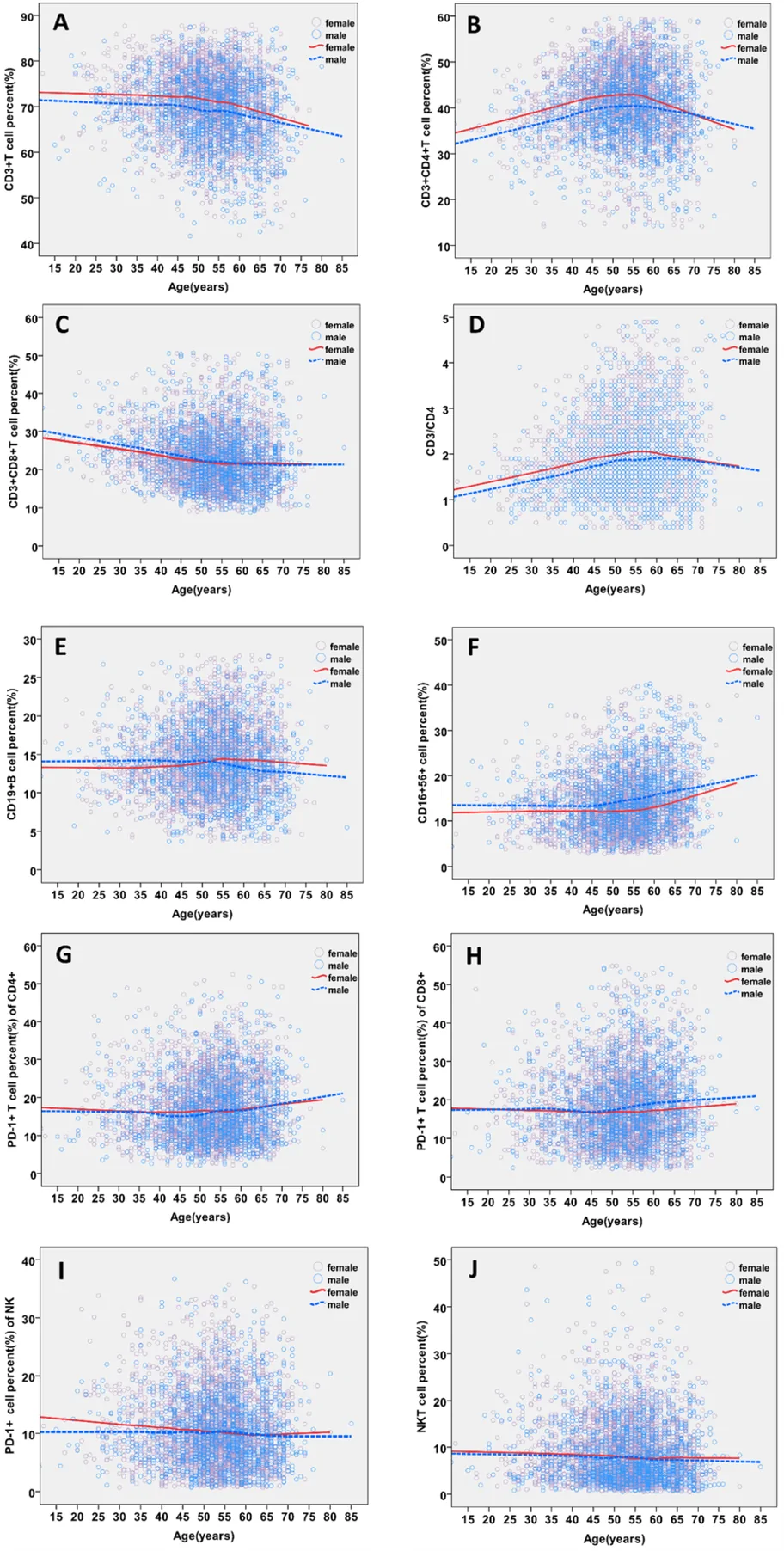
Age‐Related Trends of Lymphocyte Subsets with Sex‐Stratified Smooth Curves. Individual data points are shown for females (blue) and males (green). The sex‐stratified non‐linear trends are depicted using LOESS smooth curves (female: solid red line; male: dashed red line).

### 3.3 Age‐Stratified Trends of Lymphocyte Subsets

#### 3.3.1 Female Cohort

The age‐stratified distributions of all lymphocyte subsets for the female cohort are detailed in Table 2. Visual representations of these trends are provided in Figure 2. A significant decline in total CD3^+^ T cells (%) was observed with advancing age (*p* < 0.001). Analysis of T cell subsets revealed divergent trajectories: CD3^+^CD4^+^ T helper cells (%) increased from young adulthood to a peak in the 55–59 years group (*p* < 0.001), whereas CD3^+^CD8^+^ cytotoxic T cells (%) demonstrated a consistent decrease (*p* < 0.001). Consequently, the CD4/CD8 ratio rose significantly, reaching its zenith in the 55–59 years group (*p* < 0.001). A significant age‐related increase was also observed in CD19^+^ B cells (%) (*p* < 0.001). The expression of the exhaustion marker PD‐1 varied by cell lineage. The proportion of CD8^+^PD‐1^+^ T cells exhibited a transient peak in the 60–64 years group, though the overall trend across ages was not statistically significant (*p* = 0.180). In contrast, CD4^+^PD‐1^+^ T cells (%) showed a significant gradual increase with age (*p* = 0.039). Within the innate immune compartment, CD16^+^56^+^ NK cells (%) increased markedly, particularly in later life (*p* < 0.001). Conversely, the proportion of NK cells expressing PD‐1 (NK^+^PD‐1^+^) decreased significantly with age (*p* = 0.001). No significant age‐related trend was found for NKT cells (%) (*p* = 0.109).

**Table 2:** Distribution of immune cell subsets across age groups in females.

Immune cell subsets	18–39 (*n*)	40–44 (*n*)	45–49 (*n*)	50–54 (*n*)	55–59 (*n*)	60–64 (*n*)	65–69 (*n*)	≥ 70 (*n*)	Total (*n*)	*P*	*ε* ^*2*^
CD3^+^T cells (% Lymphocytes)	72.20 (251)	73.00 (256)	72.50 (402)	71.50 (673)	71.10 (719)	69.60 (322)	68.40 (222)	66.80 (37)	71.20 (2882)	< 0.001	0.023
CD3^+^CD4^+^T cells (% Lymphocytes)	39.20 (251)	42.10 (251)	42.40 (402)	42.60 (664)	42.80 (704)	40.60 (315)	39.90 (219)	36.70 (37)	41.90 (2843)	< 0.001	0.025
CD3^+^CD8^+^T cells (% Lymphocytes)	24.10 (251)	23.15 (258)	22.10 (405)	21.80 (674)	21.20 (706)	21.50 (317)	21.75 (218)	19.90 (37)	22.10 (2866)	< 0.001	0.018
CD4/CD8 Ratio	1.70 (249)	1.80 (251)	1.90 (401)	1.90 (670)	2.00 (702)	1.90 (316)	1.90 (213)	1.85 (36)	1.90 (2838)	< 0.001	0.020
CD19^+^B cells (% Lymphocytes)	13.10 (251)	13.40 (257)	13.45 (400)	14.20 (671)	14.20 (707)	14.00 (315)	14.10 (215)	13.30 (38)	13.90 (2854)	< 0.001	0.007
CD16^+^56^+^cells (% Lymphocytes)	12.40 (249)	11.40 (255)	11.70 (402)	12.00 (672)	12.20 (718)	13.65 (320)	14.20 (223)	20.10 (37)	12.30 (2876)	< 0.001	0.017
PD‐1^+^ (% of CD4^+^ T cells)	15.90 (251)	15.00 (257)	16.20 (404)	16.15 (678)	15.30 (720)	16.50 (318)	16.70 (222)	18.60 (38)	15.90 (2888)	0.039	0.003
PD‐1^+^ (% of CD8^+^ T cells)	16.90 (251)	16.30 (255)	16.00 (403)	16.75 (676)	16.20 (719)	18.25 (320)	16.30 (223)	19.05 (38)	16.60 (2885)	0.180	0.001
PD‐1^+^ (% of NK cells)	11.10 (249)	10.70 (253)	10.10 (404)	10.10 (669)	9.80 (712)	8.80 (321)	9.00 (221)	12.00 (38)	10.00 (2867)	0.001	0.006
NKT (% of Lymphocytes)	7.70 (252)	7.79 (258)	7.30 (406)	7.10 (676)	6.93 (720)	7.15 (322)	7.54 (223)	5.20 (38)	7.20 (2895)	0.109	0.002

*Note:* Data are presented as median (*n*), where *n* is the sample size for each age group. *ε* ^2^ was calculated as H/ (N − 1), where N is the total sample size for each immune subset (see “Total” column). For *ε* ^2^, 0.01, 0.06, and 0.14 indicate small, medium, and large effects, respectively.

**Figure 2:**
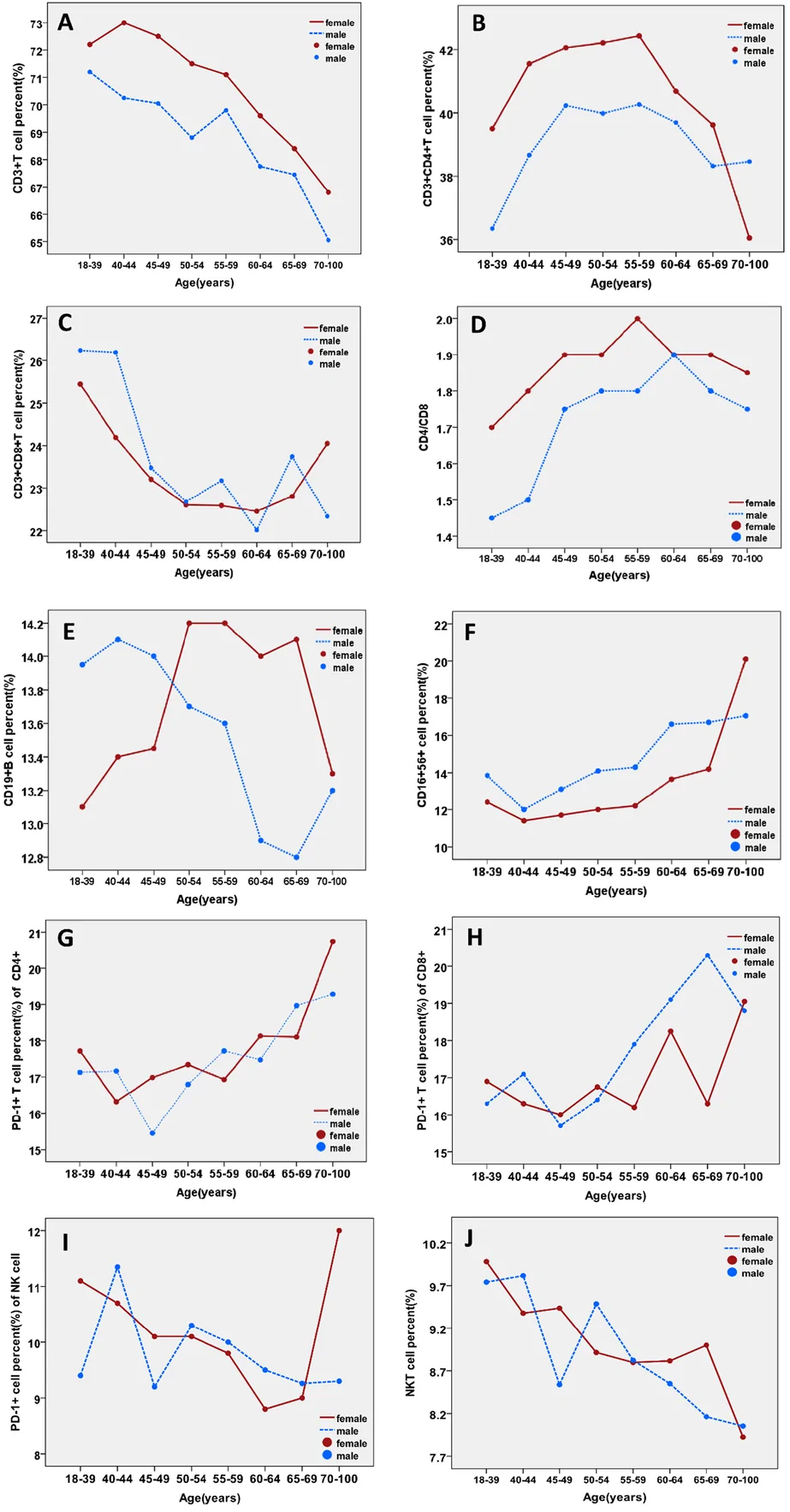
Sex differences in median levels of lymphocyte subsets across age groups. Data points and connecting lines represent the median percentage of each lymphocyte subset within each age group. Female data are connected by a solid red line, and male data by a dashed green line.

#### 3.3.2 Male Cohort (Comparative Control)

The comprehensive dataset for the male cohort is available in Supporting Information S1: Table S2. Similar to females, males exhibited significant age‐related declines in CD3^+^ T cells (%) and CD3^+^CD8^+^ T cells (%), and a significant increase in the CD4/CD8 ratio (all *p* < 0.001). However, the male CD3^+^CD4^+^ T cell percentage peaked earlier (55–59 years) and the CD8^+^PD‐1^+^ T cell percentage showed a more consistent, though not statistically significant, upward trend with age (*p* = 0.054).

### 3.4 Comparison of Lymphocyte Subsets Between Sexes After Age‐Matching

To control for the confounding effect of age, a 1:1 propensity score matching was performed, creating a balanced cohort for comparative analysis. The distributions of key lymphocyte subsets for the age‐matched male and female cohorts are presented in Table 3 and visually summarized in Figure 3. After matching, the median of CD3^+^ T cells (%) was higher in females than in males (*p* < 0.001). Analysis of T cell subsets revealed that females had a higher median of CD3^+^CD4^+^ T cells (%) (*p* < 0.001) and a lower median of CD3^+^CD8^+^ T cells (%) (*p* < 0.001). Consequently, the CD4/CD8 ratio was substantially higher in females (*p* < 0.001). Significant sex‐based differences were also observed in other lineages. The median of CD19^+^ B cells (%) was higher in females than in males (*p* < 0.001). Notably, age‑stratified analysis using unmatched raw data (Supporting Information S1: Table S3) revealed that this difference was not significant before age 50 but emerged after menopause (after age 55). Similarly, females showed a higher median of CD16^+^56^+^ NK cells (%) (*p* < 0.001), while the median of NKT cells (%) was lower in males compared to females (*p* < 0.001). The expression of the immune checkpoint PD‐1 also differed. A lower proportion of PD‐1^+^ CD4^+^ T cells (%) was found in females (*p* < 0.001). Conversely, a higher proportion of PD‐1^+^ CD8^+^ T cells (%) was observed in females (*p* < 0.001). The proportion of PD‐1^+^ NK cells (%) was lower in females than in males (*p* < 0.001). No consistent sex differences were observed beyond 70 years in the age‑stratified analysis (Supporting Information S1: Table S3).

**Table 3:** Comparison of immune cell subsets between sexes after age propensity score matching.

Immune cell subset	Female		Male		Median Difference (95% CI)	Effect size (r)	*P*
	Median (Q1‐Q3)	Matched *n*	Median (Q1‐Q3)	Matched *n*			
CD3^+^T cells (% lymphocytes)	76.30 (72.90‐79.30)	1296	69.10 (63.62‐74.30)	1296	7.90 (7.40 to 8.40)	0.573	< 0.001
CD3^+^CD4^+^T cells (% lymphocytes)	47.20 (44.60‐50.50)	1289	39.40 (34.40‐44.65)	1289	8.30 (7.30 to 8.80)	0.576	< 0.001
CD3^+^CD8^+^T cells (% lymphocytes)	17.50 (15.10‐19.50)	1300	22.60 (17.90‐27.77)	1300	−5.50 (−5.90 to −5.00)	0.463	< 0.001
CD4/CD8 ratio	2.60 (2.30‐3.10)	1285	1.80 (1.30‐2.40)	1285	0.90 (0.80 to 0.90)	0.528	< 0.001
CD19^+^B cells (% lymphocytes)	17.40 (15.80‐19.80)	1273	13.50 (10.70‐16.90)	1273	4.30 (4.00 to 4.60)	0.499	< 0.001
CD16^+^56^+^cells (% lymphocytes)	17.40 (15.00‐21.30)	1294	14.30 (10.20‐19.50)	1294	3.80 (3.30 to 4.20)	0.308	< 0.001
PD‐1^+^ (% of CD4^+^ T cells)	10.40 (8.30‐12.50)	1304	15.75 (10.90‐22.30)	1304	−5.70 (−6.20 to −5.27)	0.469	< 0.001
PD‐1^+^ (% of CD8^+^ T cells)	24.30 (20.60‐29.60)	1300	17.60 (11.80‐25.00)	1300	7.30 (6.70 to 7.90)	0.394	< 0.001
PD‐1^+^ (% of NK cells)	5.87 (3.80‐7.50)	1290	9.84 (6.30‐14.50)	1290	−4.35 (−4.70 to −4.00)	0.478	< 0.001
NKT (% of lymphocytes)	13.30 (10.20‐17.55)	1309	6.71 (3.90‐12.40)	1309	6.20 (5.89 to 6.60)	0.485	< 0.001

*Note:* Median differences (female minus male) were estimated using the Hodges–Lehmann method. Effect size r (Z/√N) indicates the magnitude of the sex difference, with values of 0.1, 0.3, and 0.5 considered small, medium, and large effects, respectively.

**Figure 3:**
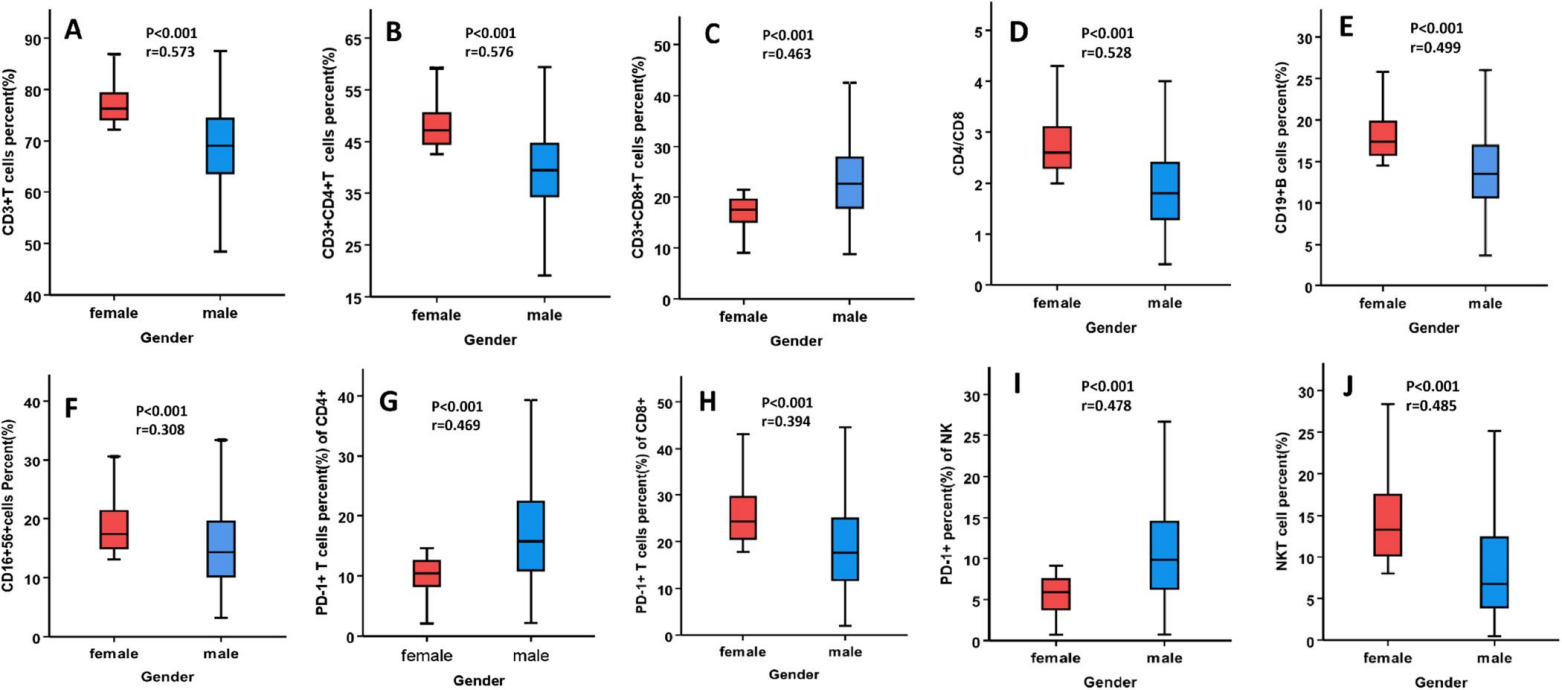
Sex differences in the distribution of lymphocyte subsets in the age‐matched cohort. Box plots show the distribution of lymphocyte subsets after 1:1 propensity score matching for age. Data for females and males are shown in red and blue, respectively.

## 4 Discussion

The reported effects of age on peripheral blood immune markers vary across studies [17, 18, 19, 20, 21, 22]. The results may be inconsistent due to different characteristic markers, variations in inclusion and exclusion criteria, and sample sizes, leading to divergent trends across studies [23]. Thus, direct comparisons between studies require careful consideration of methodological context. To delineate age‐related changes in lymphocyte subsets with a particular focus on sex differences and the menopausal transition, we conducted a systematic analysis in a large Chinese cohort. The results revealed marked differences in immune profiles between males and females. The peri‐ to early post‐menopausal period in women was identified as a critical phase of significant immune alterations, characterized by a “scissor‐like” shift in T‐cell subsets and opposite trends in B‐cell dynamics compared to males.

Consistent with earlier studies, females exhibit higher levels of CD3^+^ T cells (%), CD3^+^CD4^+^ T cells (%), and a markedly elevated CD4/CD8 ratio [24, 25, 26, 27]. Notably, these differences remain robust after adjusting for age, emphasizing their intrinsic sex‐related nature. Particularly striking is the opposing age‐related trend of CD19^+^ B cells (%), a positive association with age in females, contrasted with a negative association in males. This pronounced dimorphism suggests an essential role for sex hormones in regulating B‐cell homeostasis. Sex‐associated differences in B‐cell function have been linked to X‐chromosome–encoded immune genes and hormonal regulation [28, 29]. The X chromosome harbors numerous immune‐related genes, including those encoding Toll‐like receptors, cytokine receptors, and key regulators of T‐ and B‐cell function [30]. In parallel, the suppressive effects of androgens on B cells and adaptive immunity, as well as their age‐related decline, may further contribute to the observed patterns [31, 32]. The sustained B‐cell proportions observed in middle‐aged and older women may therefore represent a distinctive adaptive feature of female immunosenescence, although its implications for humoral immunity require further investigation.

Age‐stratified analyses of female T‐cell subsets revealed that CD3^+^CD4^+^ T cells (%) and the CD4/CD8 ratio peak at 55–59 years, whereas CD3^+^CD8^+^ cytotoxic T cells (%) show a continual decline. This “scissor‐like” pattern closely parallels the abrupt reduction in estrogen levels around menopause [33]. The timing of the peak CD4/CD8 ratio around 55–59 years coincides with the typical age of menopause, raising the possibility that declining estrogen levels may influence T‐cell subset distribution. However, as hormone levels were not measured in this study, this mechanistic interpretation remains speculative and requires direct testing in future investigations. The continuous reduction in CD8^+^ T cells, particularly cytotoxic subsets, likely reflects thymic involution, reduced naïve T‐cell output, and the higher susceptibility of CD8^+^ T cells to exhaustion and apoptosis [34, 35].

PD‐1 expression exhibited a complex, cell‐type‐specific pattern governed by both age and sex. Consistent with a previous report suggesting a male‐predominant, age‐related increase in PD‐1^+^ T cells, our correlation analysis showed numerically higher coefficients in males (*r* = 0.088 for both subsets) than in females (r = 0.033 and 0.022, respectively). Although females generally display stronger immune activation, PD‐1 expression on CD4^+^ T cells and NK cells was lower in females, potentially due to estrogen‐mediated suppression via ERα/ERβ signaling [36]. In contrast, PD‐1 expression on CD8^+^ T cells was higher in females and fluctuated with age, consistent with estrogen‐driven CD8^+^ T‐cell activation and differentiation, as well as the increased likelihood of female CD8^+^ T cells entering an exhaustion‐prone PD‐1 high state [37]. The transient peak of CD8^+^PD‐1^+^ T cells (%) at 60–64 years may mark a critical window of heightened functional dysregulation in cytotoxic immunity. The upregulation of PD‐1 on activated CD4+ and CD8 + T cells may contribute to differentiation and homeostasis of activated T cells, resulting in apoptosis and growth restriction [38]. Critically, in the context of cancer, PD‐1 expression on effector T cells is known to deplete anti‐tumor immune function and impair control of tumor growth [39]. Preoperative PD‐1^+^CD4^+^ T cells (as a percentage of total CD4^+^ T cells) were significantly associated with increased postoperative infectious complications (PIC) risk and markers of immunological and physical frailty [40]. These age‐ and sex‐specific PD‐1 patterns may have implications for the interpretation of immune checkpoint status in different demographic groups, although their clinical relevance requires prospective validation.

Several limitations should be noted. Cross‐sectional data can reveal age‐related differences but cannot track within‐individual immune changes over time. The lack of menstrual records and hormone measurements meant that formal STRAW + 10 staging was not feasible; instead, age groups were used as a pragmatic approximation. This approach broadly aligns with the major reproductive stages across most age ranges, although the 50–54 year group inevitably mixes perimenopausal and early postmenopausal women. Consequently, any interpretation of the immune patterns in terms of estrogen effects remains conjectural without direct hormonal evidence. Longitudinal studies incorporating both hormone monitoring and systematic staging are needed to move beyond these constraints.

In summary, this study demonstrates that the menopausal transition is accompanied by substantial reconfiguration of lymphocyte subsets, and that sex differences in immunosenescence are already evident in middle‐aged adults. These findings underscore the need to consider both sex and reproductive stage in future studies of immune aging. Such an approach deepens mechanistic understanding of age‐related immune alterations and provides a scientific foundation for the development of sex‐specific immunological interventions, including the optimization of vaccination strategies for middle‐aged women.

## Author Contributions

**Ci Xuan:** writing – original draft. **Li He:** data curation. **Zhou Bohan:** visualization, software, formal analysis, investigation. **Xu Junming:** visualization, software, formal analysis. **Li Qingchao:** investigation, data curation, resources. **Huang Junting:** conceptualization, methodology, funding acquisition, project administration.

## Conflicts of Interest

The authors declare no conflicts of interest.

## Supporting information


Supporting File 1

Supporting File 2

## Data Availability

The data that support the findings of this study are available from the corresponding author, Huang Junting, upon reasonable request. Due to privacy and ethical restrictions, the data are not publicly available.
