# Supplementary material for: Dynamic Shifts in Lymphocyte Subsets During the Menopausal Transition and Sexual Dimorphism in Immunosenescence
Source: Immun Inflamm Dis. 2026 Oct 15;14(10):e70558. doi: 10.1002/iid3.70558 (PMC13671003; doi:10.1002/iid3.70558)
Supplement: Supplementary file 1 [file IID3-14-e70558-s001.docx]

| Immune Cell Subsets | Female | | Male | |
| --- | --- | --- | --- | --- |
|  | **R (*P*)** | **β** | **R (*P*)** | **β** |
| CD3^+^T cells (% Lymphocytes) | -0.152(<0.001) | -0.157 | -0.135(<0.001) | -0.121 |
| CD3^+^CD4^+^T cells (% Lymphocytes) | 0.043(0.125) | 0.045 | -0.014(0.456) | -0.018 |
| CD3^+^CD8^+^T cells (% Lymphocytes) | -0.116(<0.001) | -0.117 | -0.150(<0.001) | -0.147 |
| CD4/CD8 Ratio | 0.088(<0.001) | 0.086 | 0.129(<0.001) | 0.128 |
| CD19^+^B cells (% Lymphocytes) | 0.070(<0.001) | 0.070 | -0.110(<0.001) | -0.103 |
| CD16^+^56^+^cells (% Lymphocytes) | 0.106(<0.001) | 0.110 | 0.185(<0.001) | 0.167 |
| PD-1⁺ (% of CD4⁺ T cells) | 0.033(0.080) | 0.034 | 0.088(0.001) | 0.082 |
| PD-1⁺ (% of CD8⁺ T cells) | 0.022(0.240) | 0.023 | 0.088(0.001) | 0.086 |
| PD-1⁺ (% of NK cells) | -0.074(<0.001) | -0.076 | -0.025(0.370) | -0.021 |
| NKT (% of Lymphocytes) | -0.053(0.004) | -0.054 | -0.059(0.032) | -0.056 |

**Table S1. Correlation Coefficients (r) and Regression Slopes (β) of Immune Cell Subsets with Age by Sex**

Table S2. Distribution of Immune Cell Subsets Across Age Groups in Males

| Immune Cell Subsets | 18-39(n) | 40-44(n) | 45-49(n) | 50-54(n) | 55-59(n) | 60-64(n) | 65-69(n) | ≥70(n) | Total(n) | *P* | *ε^2^* |
| --- | --- | --- | --- | --- | --- | --- | --- | --- | --- | --- | --- |
| CD3^+^T cells (% Lymphocytes) | 71.20(129) | 70.25(104) | 70.05(184) | 68.80(236) | 69.80(277) | 67.75(188) | 67.45(136) | 65.05(42) | 69.10(1296) | <0.001 | 0.016 |
| CD3^+^CD4^+^T cells (% Lymphocytes) | 37.20(129) | 38.90(104) | 39.20(182) | 40.05(234) | 40.75(276) | 40.60(185) | 38.50(135) | 37.95(44) | 39.40(1289) | <0.001 | 0.017 |
| CD3^+^CD8^+^T cells (% Lymphocytes) | 25.60(131) | 24.70(105) | 23.25(184) | 22.20(238) | 22.30(279) | 20.85(186) | 22.20(135) | 21.30(42) | 22.60(1300) | <0.001 | 0.031 |
| CD4/CD8 Ratio | 1.45(130) | 1.50(104) | 1.75(182) | 1.80(236) | 1.80(274) | 1.90(180) | 1.80(135) | 1.74(44) | 1.80(1285) | <0.001 | 0.032 |
| CD19^+^B cells (% Lymphocytes) | 13.95(130) | 14.10(103) | 14.00(178) | 13.70(232) | 13.60(273) | 12.90(183) | 12.80(131) | 13.20(43) | 13.50(1273) | 0.006 | 0.010 |
| CD16^+^56^+^cells (% Lymphocytes) | 13.85(130) | 12.00(105) | 13.10(182) | 14.10(236) | 14.30(279) | 16.60(187) | 16.70(133) | 17.05(44) | 14.30(1294) | <0.001 | 0.033 |
| PD-1⁺ (% of CD4⁺ T cells) | 14.80(131) | 15.20(104) | 14.50(183) | 15.10(238) | 16.40(278) | 15.20(190) | 17.80(137) | 17.70(43) | 15.75(1304) | 0.007 | 0.010 |
| PD-1⁺ (% of CD8⁺ T cells) | 16.30(131) | 17.10(105) | 15.70(182) | 16.40(237) | 17.90(278) | 19.10(188) | 20.30(136) | 18.80(43) | 17.60(1300) | 0.054 | 0.005 |
| PD-1⁺ (% of NK cells) | 9.40(128) | 11.35(102) | 9.20(181) | 10.30(236) | 10.00(277) | 9.50(188) | 9.26(135) | 9.30(43) | 9.84(1290) | 0.226 | 0.002 |
| NKT (% of Lymphocytes) | 7.09(131) | 7.20(105) | 6.47(185) | 7.65(238) | 6.47(281) | 6.30(189) | 6.03(136) | 6.69(44) | 6.71(1309) | 0.245 | 0.002 |

Data are presented as median (n), where n is the sample size for each age group. ε² was calculated as H/(N − 1), where N is the total sample size for each immune subset (see “Total” column). For ε², 0.01, 0.06, and 0.14 indicate small, medium, and large effects, respectively

Table S3. Sex Comparison of Immune Cell Subsets Across Age Groups (Unmatched Data)

| Age Group | Immune Subset | Female Median (Q1-Q3) | Male Median (Q1-Q3) | Median Diff (95% CI) | r | *P* |
| --- | --- | --- | --- | --- | --- | --- |
| 18-39 | CD3+T cells | 72.2(68.3 to 76.8) | 71.2(65.4 to 75.2) | 2.20(0.6 to 3.7) | 0.146 | 0.004 |
| 18-39 | CD3+CD4+T cells | 39.2(35.4 to 43.7) | 37.2(31.6 to 40.9) | 2.9(1.4 to 4.4) | 0.198 | <0.001 |
| 18-39 | CD3+CD8+T cells | 24.1(21.3 to 29.7) | 25.6(21.0 to 30.3) | -0.8(-2.3 to 0.6) | - | 0.263 |
| 18-39 | CD4/CD8 Ratio | 1.7(1.3 to 2.0) | 1.5(1.1 to 1.9) | 0.2(0.0 to 0.3) | 0.132 | 0.010 |
| 18-39 | CD19+B cells | 13.1(10.6 to 15.9) | 14.0(11.6 to 16.4) | -0.9(-1.7 to -0.1) | 0.108 | 0.036 |
| 18-39 | CD16+56+cells | 12.4(8.4 to 15.8) | 13.9(9.4 to 18.3) | -1.4(-2.6 to -0.2) | 0.119 | 0.021 |
| 18-39 | PD-1⁺ (% of CD4⁺ T cells) | 15.9(10.9 to 23.3) | 14.8(10.0 to 22.7) | 0.6(-1.1 to 2.4) | - | 0.455 |
| 18-39 | PD-1⁺ (% of CD8⁺ T cells) | 16.9(11.4 to 23.4) | 16.3(10.8 to 24.6) | 0.18(-1.7 to 2.0) | - | 0.880 |
| 18-39 | PD-1⁺ (% of NK cells) | 11.1(7.1 to 17.1) | 9.4(6.3 to 13.5) | 1.8(0.5 to 3.1) | 0.141 | 0.006 |
| 18-39 | NKT | 7.7(4.6 to 14.0) | 7.1(4.2 to 13.6) | 0.3(-0.9 to 1.4) | - | 0.637 |
| 40-44 | CD3+T cells | 73.0(66.7 to 77.3) | 70.3(67.3 to 75.1) | 1.6(0.0 to 3.1) | - | 0.051 |
| 40-44 | CD3+CD4+T cells | 42.1(36.7 to 46.1) | 38.9(33.2 to 44.4) | 2.9(1.3 to 4.6) | 0.179 | 0.001 |
| 40-44 | CD3+CD8+T cells | 23.2(19.3 to 27.4) | 24.7(21.1 to 30.3) | -2(-3.5 to -0.4) | 0.129 | 0.014 |
| 40-44 | CD4/CD8 Ratio | 1.8(1.4 to 2.4) | 1.5(1.2 to 2.1) | 0.3(0.1 to 0.4) | 0.187 | <0.001 |
| 40-44 | CD19+B cells | 13.4(10.7 to 16.3) | 14.1(10.9 to 17.7) | -0.7(-1.8 to 0.3) | - | 0.167 |
| 40-44 | CD16+56+cells | 11.4(8.3 to 16.9) | 12.0(9.0 to 17.2) | -0.6(-1.9 to 0.7) | - | 0.348 |
| 40-44 | PD-1⁺ (% of CD4⁺ T cells) | 15.0(10.8 to 21.1) | 15.2(11.3 to 22.2) | -0.6(-2.4to1.1) | - | 0.455 |
| 40-44 | PD-1⁺ (% of CD8⁺ T cells) | 16.3(11.8 to 21.8) | 17.1(12.0 to 22.6) | -0.9(-2.8 to 1.0) | - | 0.374 |
| 40-44 | PD-1⁺ (% of NK cells) | 10.7(6.2 to 15.4) | 11.4(7.0 to 16.6) | -0.9(-2.3 to 0.6) | - | 0.266 |
| 40-44 | NKT | 7.8(4.5 to 12.5) | 7.2(3.8 to 13.6) | 0.2(-1.0 to 1.4) | - | 0.725 |
| 45-49 | CD3+T cells | 72.5(67.7 to 76.8) | 70.1(63.9 to 75.3) | 2.4(1.1 to 3.8) | 0.143 | 0.001 |
| 45-49 | CD3+CD4+T cells | 42.4(37.9 to 46.7) | 39.2(35.3 to 44.7) | 2.3(1.0 to 3.6) | 0.145 | <0.001 |
| 45-49 | CD3+CD8+T cells | 22.1(18.3 to 27.2) | 23.3(17.0 to 27.6) | -0.1(-1.4 to 1.1) | - | 0.843 |
| 45-49 | CD4/CD8 Ratio | 1.9(1.5 to 2.4) | 1.8(1.4 to 2.4) | 0.1(0.0 to 0.2) | - | 0.124 |
| 45-49 | CD19+B cells | 13.5(11.1 to 16.5) | 14.0(11.4 to 17.4) | -0.6(-1.4 to 0.2) | - | 0.120 |
| 45-49 | CD16+56+cells | 11.7(8.2 to 16.0) | 13.1(9.2 to 17.5) | -1.1(-2.1 to 0.0) | 0.085 | 0.041 |
| 45-49 | PD-1⁺ (% of CD4⁺ T cells) | 16.2(10.4 to 21.8) | 14.5(9.7 to 18.9) | 1.6(0.3 to 2.8) | 0.097 | 0.019 |
| 45-49 | PD-1⁺ (% of CD8⁺ T cells) | 16.0(10.4 to 22.6) | 15.7(10.8 to 22.9) | -0.2(-1.7 to 1.3) | - | 0.812 |
| 45-49 | PD-1⁺ (% of NK cells) | 10.1(6.7 to 14.4) | 9.2(5.8 to 13.9) | 0.9(-0.14 to 1.9) | - | 0.093 |
| 45-49 | NKT | 7.3(4.1 to 13.6) | 6.5(3.6 to 11.3) | 0.7(-0.1 to 1.6) | - | 0.08 |
| 50-54 | CD3+T cells | 71.5(66.7 to 75.8) | 68.8(63.9 to 73.9) | 2.3(1.2 to 3.4) | 0.134 | <0.001 |
| 50-54 | CD3+CD4+T cells | 42.6(38.2 to 47.3) | 40.1(35.4 to 44.6) | 2.4(1.4 to 3.5) | 0.147 | <0.001 |
| 50-54 | CD3+CD8+T cells | 21.8(17.8 to 26.3) | 22.2(18.0 to 26.7) | -0.1(-1.1 to 0.9) | - | 0.794 |
| 50-54 | CD4/CD8 Ratio | 1.9(1.5 to 2.6) | 1.8(1.4 to 2.4) | 0.1(0.0 to 0.2) | 0.066 | 0.046 |
| 50-54 | CD19+B cells | 14.2(11.3 to 17.2) | 13.7(11.3 to 17.1) | 0.3(-0.4 to 0.9) | - | 0.438 |
| 50-54 | CD16+56+cells | 12.0(8.7 to 16.6) | 14.1(10.5 to 19.5) | -2.1(-3.0 to -1.2) | 0.151 | <0.001 |
| 50-54 | PD-1⁺ (% of CD4⁺ T cells) | 16.2(11.0 to 23.0) | 15.1(10.9 to 22.2) | 0.6(-0.6 to 1.7) | - | 0.339 |
| 50-54 | PD-1⁺ (% of CD8⁺ T cells) | 16.8(11.3 to 23.7) | 16.4(11.5 to 24.3) | 0.4(-1.8 to 0.9) | - | 0.513 |
| 50-54 | PD-1⁺ (% of NK cells) | 10.1(6.1 to 15.0) | 10.3(6.6 to 14.7) | -0.2(-1.1 to 0.7) | - | 0.681 |
| 50-54 | NKT | 7.1(4.0 to 12.4) | 7.7(4.3 to 12.9) | -0.4(-1.2 to 0.4) | - | 0.295 |
| 55-59 | CD3+T cells | 71.1(66.3 to 75.6) | 69.8(64.2 to 74.0) | 1.4(0.4 to 2.4） | 0.085 | 0.008 |
| 55-59 | CD3+CD4+T cells | 42.8(37.7 to 47.5) | 40.8(35.2 to 46.2) | 2.2(1.1 to 3.3) | 0.127 | <0.001 |
| 55-59 | CD3+CD8+T cells | 21.2(17.0 to 26.7) | 22.3(17.9 to 27.3) | -0.7(-1.7 to 0.2) | - | 0.136 |
| 55-59 | CD4/CD8 Ratio | 2.0(1.5 to 2.7) | 1.8(1.3 to 2.5) | 0.2(0.1 to 0.3) | 0.101 | 0.002 |
| 55-59 | CD19+B cells | 14.2(11.7 to 17.5) | 13.6(10.5 to 17.3) | 0.7(0.1 to 1.4) | 0.069 | 0.031 |
| 55-59 | CD16+56+cells | 12.2(8.8 to 16.0) | 14.3(10.4 to 19.0) | -2.1(-2.9 to -1.3) | 0.159 | <0.001 |
| 55-59 | PD-1⁺ (% of CD4⁺ T cells) | 15.3(10.7 to 22.0) | 16.4(11.4 to 22.3) | -0.9(-2.0 to 0.2) | - | 0.111 |
| 55-59 | PD-1⁺ (% of CD8⁺ T cells) | 16.2(10.8 to 23.1) | 17.9(12.4 to 35.2) | -1.8(-3.1 to -0.5) | 0.087 | 0.006 |
| 55-59 | PD-1⁺ (% of NK cells) | 9.8(6.3 to 14.1) | 10.0(7.0 to 14.8) | -0.2(-1.0 to 0.6) | - | 0.620 |
| 55-59 | NKT | 6.9(3.8 to 12.1) | 6.5(4.0 to 12.4) | 0.0(-0.7 to 0.7) | - | 0.960 |
| 60-64 | CD3+T cells | 69.6(63.8 to 74.9) | 67.8(61.5 to 74.1) | 1.9(0.3 to 3.4) | 0.107 | 0.016 |
| 60-64 | CD3+CD4+T cells | 40.6(35.4 to 45.1) | 40.6(32.6 to 46.5) | 0.8(-0.8 to 2.4 | - | 0.335 |
| 60-64 | CD3+CD8+T cells | 21.5(17.4 to 26.8) | 20.9(16.1 to 25.8) | 0.9(-0.3 to 2.2) | - | 0.153 |
| 60-64 | CD4/CD8 Ratio | 1.9(1.4 to 2.5) | 1.9(1.4 to 2.6) | 0.0(-0.2 to 0.1) | - | 0.828 |
| 60-64 | CD19+B cells | 14.0(11.2 to 17.1) | 12.9(9.4 to 16.5) | 1.2(0.3 to 2.1) | 0.120 | 0.008 |
| 60-64 | CD16+56+cells | 13.7(9.3 to 18.9) | 16.6(11.4 to 22.7) | -2.7(-4.0 to -1.5) | 0.167 | <0.001 |
| 60-64 | PD-1⁺ (% of CD4⁺ T cells) | 16.5(11.3 to 24.4) | 15.2(9.8 to 23.5) | 1.0(-0.5 to 2.5) | - | 0.172 |
| 60-64 | PD-1⁺ (% of CD8⁺ T cells) | 18.3(11.7 to 24.3) | 19.1(11.8 to 27.9) | -1.5(-3.3 to 0.3) | - | 0.103 |
| 60-64 | PD-1⁺ (% of NK cells) | 8.8(5.0 to 14.8) | 9.5(5.6 to 14.4) | -0.2(-1.4 to 0.9) | - | 0.677 |
| 60-64 | NKT | 7.2(3.4 to 12.2) | 6.3(3.7 to 11.7) | 0.2(-0.7 to 1.1) | - | 0.608 |
| 65-69 | CD3+T cells | 68.4(63.6 to 74.6) | 67.5(60.3 to 72.7) | 1.6(-0.2 to 3.5) | - | 0.078 |
| 65-69 | CD3+CD4+T cells | 39.9(34.6 to 45.5) | 38.5(32.9 to 44.3) | 1.4(-0.5 to 3.2) | - | 0.151 |
| 65-69 | CD3+CD8+T cells | 21.8(17.6 to 27.4) | 22.2(16.5 to 27.2) | -0.2(-1.9 to 1.3) | - | 0.791 |
| 65-69 | CD4/CD8 Ratio | 1.9(1.4 to 2.5) | 1.8(1.4 to 2.4) | 0.1(-0.1 to 0.3) | - | 0.413 |
| 65-69 | CD19+B cells | 14.1(11.5 to 17.6) | 12.8(9.2 to 15.7) | 1.8(0.7 to 2.8) | 0.174 | 0.001 |
| 65-69 | CD16+56+cells | 14.2(10.1 to 18.6) | 16.7(12.3 to 23.0) | -2.2(-3.7 to 0.7) | 0.152 | 0.004 |
| 65-69 | PD-1⁺ (% of CD4⁺ T cells) | 16.7(10.7 to 24.5) | 17.8(12.0 to 24.3) | -0.9(-2.7 to 1.0) | - | 0.374 |
| 65-69 | PD-1⁺ (% of CD8⁺ T cells) | 16.3(11.2 to 23.9) | 20.3(12.8 to 27.1) | -2.9(-4.9 to 0.8) | 0.139 | 0.008 |
| 65-69 | PD-1⁺ (% of NK cells) | 9.0(5.3 to 13.9) | 9.3(5.9 to 13.5) | -0.3(-1.5 to 1.0) | - | 0.652 |
| 65-69 | NKT | 7.5(4.3 to 12.2) | 6.0(3.1 to 11.7) | 0.9(-0.2 to 2.0) | - | 0.095 |
| ≥70 | CD3+T cells | 66.8(58.1 to 73.0) | 65.1(60.6 to 75.2) | -0.7(-5.6 to 3.9) | - | 0.716 |
| ≥70 | CD3+CD4+T cells | 36.7(30.7 to 42.1) | 38.0(33.8 to 45.5) | -2.4(-6.7 to 1.8) | - | 0.253 |
| ≥70 | CD3+CD8+T cells | 19.9(15.8 to 31.7) | 21.3(17.5 to 27.4) | 0.5(-3.5 to 4.3) | - | 0.844 |
| ≥70 | CD4/CD8 Ratio | 1.9(1.3 to 2.4) | 1.8(1.2 to 2.9) | 0.0(-0.5 to 0.4) | - | 0.946 |
| ≥70 | CD19+B cells | 13.3(9.0 to 16.0) | 13.2(9.5 to 15.7) | 0.2(-1.8 to 2.3) | 0.802 | 0.027 |
| ≥70 | CD16+56+cells | 20.1(12.2 to 25.1) | 17.1(11.6 to 23.7) | 1.6(-2.9 to 5.1) | - | 0.470 |
| ≥70 | PD-1⁺ (% of CD4⁺ T cells) | 18.6(13.5 to 25.6) | 17.7(13.0 to 25.4) | 1.0(-2.7to 5.0) | - | 0.613 |
| ≥70 | PD-1⁺ (% of CD8⁺ T cells) | 19.1(13.7 to 28.4) | 18.8(13.3 to 24.8) | 0.8(-2.7 to 4.6) | - | 0.646 |
| ≥70 | PD-1⁺ (% of NK cells) | 12.0(7.1 to 16.9) | 9.3(6.7 to 14.5) | 2.3(-0.47 to 5.0) | - | 0.122 |
| ≥70 | NKT | 5.2(3.2 to 10.6) | 6.7(2.8 to 11.8) | -0.5(-2.9 to 1.8) | - | 0.639 |

Q1 and Q3 are the 25th and 75th percentiles. Median differences are female minus male. Effect size r indicates the magnitude of the sex difference, with 0.1, 0.3, and 0.5 representing small, medium, and large effects, respectively. "–" indicates P > 0.05, and thus the effect size was not calculated.
